# Supplementary material for: Micromanipulation of prophase I chromosomes from mouse spermatocytes reveals high stiffness and gel-like chromatin organization
Source: Commun Biol. 2020 Sep 30;3:542. doi: 10.1038/s42003-020-01265-w (PMC7528058; doi:10.1038/s42003-020-01265-w)
Supplement: Supplementary file 2 — Description of Additional Supplementary Files [file 42003_2020_1265_MOESM2_ESM.pdf]

Description of Additional Supplementary Files

File Name: Supplementary Data 1
Description: This spreadsheet contains the numerical data used to generate all of the data figures in the paper. Each page in the spreadsheet contains the data for one figure, as indicated.
