# Supplementary material for: Micromanipulation of prophase I chromosomes from mouse spermatocytes reveals high stiffness and gel-like chromatin organization
Source: Commun Biol. 2020 Sep 30;3:542. doi: 10.1038/s42003-020-01265-w (PMC7528058; doi:10.1038/s42003-020-01265-w)
Supplement: Supplementary file 4 — Reporting Summary [file 42003_2020_1265_MOESM4_ESM.pdf]

nature research

Corresponding author(s): *Double-blind peer review submissions: write DBPR and your manuscript number here instead of author names.*

Last updated by author(s): 2020 07 17

Reporting Summary

Nature Research wishes to improve the reproducibility of the work that we publish. This form provides structure for consistency and transparency in reporting. For further information on Nature Research policies, see our Editorial Policies and the Editorial Policy Checklist.

Statistics

For all statistical analyses, confirm that the following items are present in the figure legend, table legend, main text, or Methods section.

n/a	Confirmed	
☐	☒	The exact sample size (*n*) for each experimental group/condition, given as a discrete number and unit of measurement
☐	☒	A statement on whether measurements were taken from distinct samples or whether the same sample was measured repeatedly
☐	☒	The statistical test(s) used AND whether they are one- or two-sided<br>*Only common tests should be described solely by name; describe more complex techniques in the Methods section.*
☐	☒	A description of all covariates tested
☐	☒	A description of any assumptions or corrections, such as tests of normality and adjustment for multiple comparisons
☐	☒	A full description of the statistical parameters including central tendency (e.g. means) or other basic estimates (e.g. regression coefficient) AND variation (e.g. standard deviation) or associated estimates of uncertainty (e.g. confidence intervals)
☐	☒	For null hypothesis testing, the test statistic (e.g. *F*, *t*, *r*) with confidence intervals, effect sizes, degrees of freedom and *P* value noted<br>*Give P values as exact values whenever suitable.*
☒	☐	For Bayesian analysis, information on the choice of priors and Markov chain Monte Carlo settings
☒	☐	For hierarchical and complex designs, identification of the appropriate level for tests and full reporting of outcomes
☒	☐	Estimates of effect sizes (e.g. Cohen's *d*, Pearson's *r*), indicating how they were calculated

Our web collection on statistics for biologists contains articles on many of the points above.

Software and code

Policy information about availability of computer code

Data collection	Computer codes for data collection used were written in LabView and are available on request.
Data analysis	Computer codes for data analysis were either written in python or used standard programs (e.g., Excel) and are available on request.

For manuscripts utilizing custom algorithms or software that are central to the research but not yet described in published literature, software must be made available to editors and reviewers. We strongly encourage code deposition in a community repository (e.g. GitHub). See the Nature Research guidelines for submitting code & software for further information.

Data

Policy information about availability of data

All manuscripts must include a data availability statement. This statement should provide the following information, where applicable:
- Accession codes, unique identifiers, or web links for publicly available datasets
- A list of figures that have associated raw data
- A description of any restrictions on data availability

All raw data files from chromosome isolation (documentation images and stretch-deflection tracking files), available on request.

Field-specific reporting

Life sciences study design

All studies must disclose on these points even when the disclosure is negative.

Sample size	Stated in paper: the mechanical measurements range from 8 to 17 measurements per condition, the digestion measurements range from 3 to 4 measurements, all experiments from unique cells (biological replicates).
Data exclusions	A single data point was excluded from the mechanical measurements on Sycp1-/- strain. This was excluded as it was an outlier calculated by the Grubbs test with an alpha value of 0.01. The inclusion of the data point did not change the statistical significance threshold ($p<0.05$) between the mechanical data of the WT spermatocyte chromosomes, nor the MEF mechanical data.<br>A single Trypsin digestion run from both MEF and WT spermatocyte chromosome was excluded from analysis as both chromosomes stiffened after treatment, attributed to an appreciable amount of debris buildup from the spray.
Replication	Stated in paper.
Randomization	Not relevant to this paper.
Blinding	Not relevant to this paper.

Reporting for specific materials, systems and methods

We require information from authors about some types of materials, experimental systems and methods used in many studies. Here, indicate whether each material, system or method listed is relevant to your study. If you are not sure if a list item applies to your research, read the appropriate section before selecting a response.

Materials & experimental systems

n/a	Involved in the study	
☐	☒	Antibodies
☐	☒	Eukaryotic cell lines
☒	☐	Palaeontology and archaeology
☐	☒	Animals and other organisms
☒	☐	Human research participants
☒	☐	Clinical data
☒	☐	Dual use research of concern

Methods

n/a	Involved in the study	
☒	☐	ChIP-seq
☒	☐	Flow cytometry
☒	☐	MRI-based neuroimaging

Antibodies

Antibodies used	Described in paper.
Validation	Commercial antibodies were tested on standard targets (cells isolated from mouse testes) for expected targeting, before use in these experiments.

Eukaryotic cell lines

Policy information about cell lines

Cell line source(s)	Pre-existing cell line in lab collection.
Authentication	Untested/base cell line.
Mycoplasma contamination	Untested.
Commonly misidentified lines (See ICLAC register)	*Name any commonly misidentified cell lines used in the study and provide a rationale for their use.*

Animals and other organisms

Policy information about studies involving animals; ARRIVE guidelines recommended for reporting animal research

Laboratory animals	Described in paper.
Wild animals	N/A
Field-collected samples	N/A

Ethics oversight	Animal procedures approved by University of Illinois IRB.

Note that full information on the approval of the study protocol must also be provided in the manuscript.
